# Functional Acrylic Resins Prepared via Photo-Induced Telomerization Using Tetrabromomethane as Telogen

**DOI:** 10.3390/ma16247650

**Published:** 2023-12-14

**Authors:** Mateusz Weisbrodt, Agnieszka Kowalczyk, Beata Schmidt, Tomasz J. Idzik, Jacek G. Sośnicki

**Affiliations:** Department of Chemical Organic Technology and Polymeric Materials, Faculty of Chemical Technology and Engineering, West Pomeranian University of Technology in Szczecin, 70-322 Szczecin, Poland; mateusz.weisbrodt@zut.edu.pl (M.W.); beata.schmidt@zut.edu.pl (B.S.); tomasz.idzik@zut.edu.pl (T.J.I.); jacek.sosnicki@zut.edu.pl (J.G.S.)

**Keywords:** tetrabromomethane, telomerization, photopolymerization, acrylic resins

## Abstract

Novel method of obtaining functional acrylic resins (FARs) containing carboxyl- and benzophenone groups (in-chain functionalization) and terminal Br atoms was verified. Acrylic oligomers were prepared by a solution-free, UV-initiated telomerization process of basic monomer (n-butyl acrylate) and functional monomers (acrylic acid and 4-acrylooxybenzophenone) in the presence of radical photoinitiator and different amount of tetrabromomethane (CBr_4_) as telogen. The effect of telogen content on UV-telomerization kinetics as well as physicochemical and thermal (T_g_) properties of FARs was investigated. A telogen content higher than 5 wt. parts does not affect the UV-telomerization rate (photo-DSC), the molecular weights of telomers (GPC), or their glass transition temperature (DSC), but it significantly increases the conversion of monomers (up to 88%) and lowers the viscosity of FARs (approx. 6 Pa·s). NMR studies confirmed the inclusion of CBr_4_ in the structure of functional acrylic telomers.

## 1. Introduction

Acrylic resins (ARs) are essential products for various industrial application fields. Particularly, they play important roles as binders and polymer thickeners in the coatings industry. ARs are mainly referred to as acrylic ester prepolymers or oligomers. Owing to their exceptional chemical resistance, mechanical properties, and strong adhesion, they find extensive application in the manufacturing of paints, primers [1], wood coatings [2], additives for silicone resins [3], flexible coatings [4], synthetic mortar compounds, insulating materials [5], as well as pressure-sensitive adhesives [6,7], prepregs, and ballistic protection [8]. In the industry, ARs are classified into two main groups. i.e., “stenomeric acrylates” (from the Greek “*steno*” and “*meris*”, which means “narrow molecular weight distribution”) and “eurymeric acrylates” (from the Greek “*euru*” and “*meris*”, according to “broad molecular weight distribution”). Stenomeric acrylates are well defined and characterized by low molecular weight. They can be described by an idealized structural formula. According to this definition, popular mono- and multifunctional reactive diluents such as TMPTA (trimethylolpropane triacrylate) or HDDA (1,6-hexanediol diacrylatebelong) can be attributed to the stenomeric acrylates. Among eurymeric acrylates, there are four main classes, i.e., epoxy acrylates, polyester acrylates, polyether acrylates, and polyurethane acrylates [8]. There are also a number of other special ARs available; for example: amino functionalized acrylates (prepared by the Michael addition reaction) [9] and acrylated polyacrylates (synthesized by transesterification of pendant hydroxyl or epoxy groups with methyl acrylate or addition of acrylic acid to the epoxide, respectively). These resins are used only in niche applications, such as adhesion promoters and some outdoor applications (pressure-sensitive adhesives, PSA). Other classes are dendrimers, hyperbranched acrylates, and silicone acrylates (mono-functional tris (alkoxy) silyl acrylates and classical organopolysiloxanes). The main applications of the acrylated polysiloxanes are release coatings for pressure-sensitive adhesives or coating additives [10,11,12]. Nevertheless, the main group of functional acrylic resins are hydroxy-, -epoxy- and acrylate functional [13,14]. The synthesis and characterization of FARs have undergone remarkable advancements, driven by innovative polymerization techniques that provide precise control over their architecture. Notably, controlled/living radical polymerization methods, such as living anionic polymerization (LAP), atom transfer radical polymerization (ATRP), and reversible addition-fragmentation chain transfer (RAFT), have enabled the fine-tuning of polymer structures and functionalities [15,16]. One of the first controlled polymerization techniques was LAP, used to synthesize functionalized polymers of two types, i.e., functionalized at the end of the polymer chain (so-called chain-end functionalized polymers) or in the middle of the polymer chain (in-chain functionalize polymers). LAP requires an appropriate solvent (e.g., benzene, toluene, tetrahydrofuran), a temperature, and initiators (alkyllithium type). However, one of the methods of synthesizing polymers functionalized at the end of the chain is to terminate the living end of the chain using a functional group. It is possible to obtain polymers with terminal amino, merpactan, carboxyl or hydroxyl groups. In-chain functionalization is also possible. The simplest approach is to use functional monomers, but it is difficult to avoid reactions between these groups and the growing end of the polymer chain. In the field of anionic polymerization of acrylic monomers, n-butyl acrylate and methyl methacrylate processes using a tetrabutylammonium counterion are known. The functionalization of polymer end groups can be achieved in two ways: either deactivation of the living chain end with an appropriate electrophile or initiation of living polymerization with an appropriate functional initiator. Among the controlled polymerization methods, ATRP is the most widely described. In this technique, a catalytic system consisting of metal salts and ligands is important. The disadvantage of controlled polymers obtained thanks to ATRP is metal contamination, which disqualifies them from some applications, e.g., medical ones. A new approach is the use of reduced amounts of metal catalysts with higher catalytic efficiency. ATRP allows the acquisition of polymers with controlled macromolecular architectures that are different in terms of topology (linear, branched, star-shaped polymers, polymer networks, and cyclic polymers), functionality (functionalized ends of the polymer chain or only hanging groups, and telechelic and multifunctional polymers). Examples of functional initiators used in LAP and ATRP are 4-cyanobenzyl bromide, 4-bromobenzyl bromide, 2-bromopropionitryle, bromoacetonitrile, glycol 2-bromopropionate, tert-butyl 2-bromopropionate, and hydroxyethyl 2-bromopropionate [17]. The common feature of the functional initiators listed here is that they contain a bromine atom. The method for obtaining FARs described in this paper also uses an organic bromine compound, i.e., tetrabromomethane (CBr_4_), as a chain transfer agent in the photo-induced telomerization process (UV-telomerization). The synthetic applications involving CBr_4_ have made considerable progress in diverse organic synthesis. Tetrabromomethane is an important compound that is less expensive and stable in laboratory conditions. It can undergo homo- and heterolytic cleavage, thus forming species that in turn can act as a reagent and mediate/catalyze many organic transformations to synthesize pharmaceuticals, agrochemicals, and natural products [18,19,20]. Apart from its use as the radical source, CBr_4_ catalyzes reactions via noncovalent interactions such as halogen bonding [21]. The photolysis processes of this compound are also known [22]. Since the 1970s, it has been used as one of the most important telogen in the process of thermally initiating the radical telomerization of acrylates [23]. The developments of the photocatalytic methods with CBr_4_ are important practical features for the broad range of organic transformations.

A relatively novel method for obtaining functional acrylic resins is telomerization [24,25]. It is a chain reaction of monomer(s) and telogen in polymerization conditions, but the products are polymers/oligomers with relatively low molecular weight and dispersity [26]. This reaction can be induced by various factors, such as temperature (mainly), metal ions, UV radiation, and gamma radiation originating from the decay of cobalt (^60^Co) [27]. Telogens, which are crucial reagents, are saturated chemical compounds that should react with radicals generated from the initiator’s (or photoinitiator’s) decomposition during the initiation stage, creating new radicals that induce monomer polymerization. Telogens can be categorized into three groups. The first group comprises alkyl halides, for example tetrachloromethane [28]. Alkyl halides, particularly those containing chlorine, bromine, or iodine, are highly efficient telogens [29]. The second group includes organic compounds containing an active telomerization center linked to a carbon atom, such as alcohols, carboxylic acids, amines, etc. Due to their properties, they are typically weak telogens but good chain transfer agents [30,31,32]. The third group comprises other compounds in which a bond other than carbon-heteroatom undergoes decomposition during initiation, such as S-H, Si-H, or P-H bonds (sulfur, silicon, and phosphorus compounds) [33,34]. Nevertheless, in the field of photochemical telomerization, telogen-type effects remain poorly understood, and investigations have been restricted to bromotrichloromethane.

This paper describes new acrylic resins functionalized in-chain and containing pendant carboxyl- and benzophenone groups as well as terminal bromine atoms, i.e., functional acrylic resins (FARs). These materials were prepared via the UV-photo-induced telomerization of selected acrylic monomers and CBr_4_ as the telogen. Solutions of acrylate telomeres in unreacted monomers were obtained, i.e., telomers syrups, which here are called functional acrylic resins. These types of resins are photoreactive (due to the presence of a benzophenone groups and a relatively small amount of unreacted monomers, which also act as reactive diluents). In turn, the presence of carboxyl groups in the side chains gives the resins greater adhesion to polar substrates. The influence of the telogen on the process and the properties of the resins was studied in detail. The use of one of this type of resin has already been presented in another article [6]. Nevertheless, we present for the first time the influence of CBr_4_ as a telogen on the UV-telomerization process.

## 2. Materials and Methods

### 2.1. Materials

The following monomers (a)–(c), telogen (d), and radical photoinitiator (d) for the UV-telomerization process were used:

(a) n-butyl acrylate (BA), (BASF, Ludwigshafen, Germany),
(b) (acrylic acid (AA), (BASF, Ludwigshafen, Germany),
(c) 4-acryloylooxybenzophenone (ABP, Chemitec, Scandiccy, Italy),
(d) ethyl(2,4,6-trimethylbenzoyl)-phenyl phosphinate (Omnirad TPOL; IGM Resins, Waalwijk, The Netherlands),
(e) tetrabromomethane (CBr_4_; Merck, Warsaw, Poland).

The components were applied without purification. The structures of monomers, photoinitiator (PI), and telogen are shown in Table 1.

### 2.2. Synthesis and Characterization of Telomers Syrups

**Figure 1:**
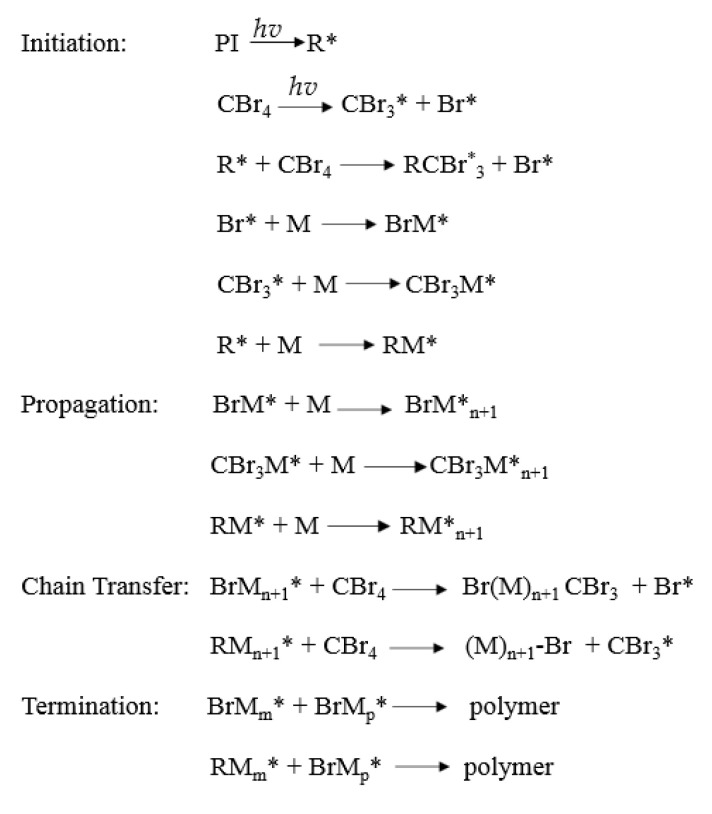
Proposed mechanism of UV-telomerization process using CBr_4_ as telogen.

**Figure 2:**
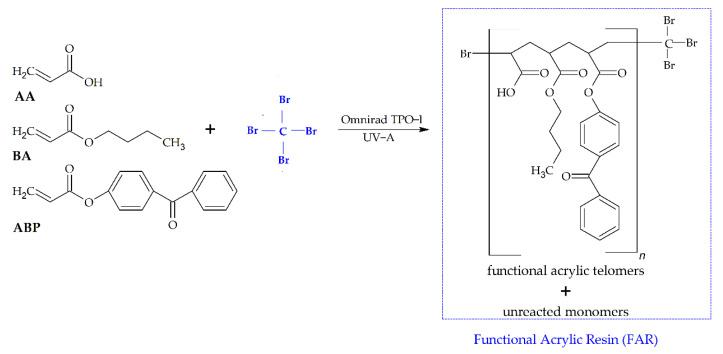
The schematic reaction of the functional acrylic resin’s preparation.

The UV-photo-induced telomerization processes of BA, AA, and ABP were initiated using radical photoinitiator TPOL, and different amounts of CBr_4_ were used. The UV-telomerization processes were carried out at 25 °C for 15 min in a glass reactor (250 mL) equipped with a mechanical stirrer, a thermocouple, a water cooler, and a capillary dosing inert gas (Ar). A high-intensity UV lamp (UVAHAND 250, Dr. Hönle AG UV Technology, Gräfelting, Germany) as a UV radiation source was used and was placed perpendicularly to the side wall of the reactor. The UV irradiation inside the reactor (15 mW/cm^2^) was controlled with UV-radiometer SL2W (UV-Design, Brachttal, Germany). The reactor was water-cooled (using room-temperature water). The temperature during the reaction was monitored. The compositions of the reaction mixtures are shown in Table 2, and the proposed mechanism of the reaction and schematic reaction are shown in Figure 1 and Figure 2, respectively.

The kinetics studies of the UV-telomerization process were realized at 25 °C by the photo-DSC method (the differential scanning calorimeter with UV equipment; Q100, TA Instruments, New Castle, DE, USA; UV-light emitter Omnicure S2000; Excelitas Technologies, Waltham, MA, USA). During the measurements, samples (5 mg) were UV-irradiated (320–390 nm) with an intensity of 15 mW/cm^2^ in argon atmosphere. Polymerization rate (*R_p_*, %/s) was calculated according to Equation (1), the conversion of double bonds (*p*, %) according to Equation (2), and photoinitiation index (*I_p_*) according to Equation (3) [29].

(1) $$R_{p}=\frac{{∆H}_{t}/dt}{H_{0}}$$

(2) $$p=\frac{{∆H}_{t}}{H_{0}}\;\times \;100\%$$

(3) $$I_{p\;}=\frac{R_{p}^{max}}{t_{max}}$$

where: *dH*/*dt*—the recorded heat flow during UV-irradiation; *H*_0_—the theoretical heat value for the complete degree of conversion (Δ*H* = 78.0 kJ/mol for acrylates); and Δ*H_t_*—the reaction heat evolved at time t.

The physicochemical properties of the telomers syrups (TS) and telomers (T) were examined. The dynamic viscosity of the TS was measured at 25 °C using the DV-II Pro Extra viscometer (spindle #6 or #7, 50 rpm; Brookfield, New York, NY, USA). The solids content (*SC*) was determined using a thermobalance (Radwag, Radom, Poland); samples (ca. 2 g) were heated in an aluminum pan at 105 °C for 4 h, and *SC* was calculated according to Equation (4):

(4) $$SC=\frac{m_{2}}{m_{1}}\cdot 100\;(wt\%)$$

where: *m*_1_—initial weight of a sample and *m*_2_—residual weight after an evaporation process. Gel permeation chromatography (GPC) was used to determine the molecular masses (Mw, Mn) and dispersity (Đ) of the acrylic telomers (post-reaction mixtures were dried at 140 °C for 4 h before the test to remove unreacted monomers); the GPC apparatus contained the refractive index detector (Merck Lachrom RI L-7490, Abingdon, UK), pump (Merck Hitachi Liquid Chromatography L-7100, Abingdon, UK) and interface (Merck Hi-tachi Liquid Chromatography D-7000, Abingdon, UK), and the Shodex Ohpak SB-806 MQ column with Shodex Ohpak SB-G pre-column (Merck Hitachi Liquid Chromatography L-7100, Abingdon, UK). The GPC tests were performed using polystyrene standards (Fluka and Polymer Standards Service GmbH, Mainz, Germany) in tetrahydrofuran. For the selected system, *SC* and molecular masses were tested as a function of the UV-telomerization time. ^1^H and ^13^C spectroscopic measurements were performed on a Bruker DPX 400 Avance III HD spectrometer operating at 400.2 and 100.6 MHz, respectively. TMS (internal standard, δ_H,C_ = 0 ppm) was used as reference, and spectra were acquired in 5 mm probes at 21 °C. For NMR analyses, MestReNova (version 12.0.3) program was used. Quantitative analyzes were performed using the internal standard, which was 2,4-dinitrobenzaldehyde. The glass transition temperature (T_g_) of the telomers was determined using differential scanning calorimetry (DSC 250; TA Instruments, New Castle, DE, USA). Samples (ca. 10 mg) of the dry telomers were placed in hermetic aluminum pans and heated from −80 °C to 200 °C at the heating rate of 10 °C/min. The T_g_ values were determined as a temperature value of the endothermic inflection point.

The K-values for the dry telomers were determined using an Ubbelohde viscometer according to the EN ISO 1628-1:1998 standard [35] and the Fikentscher equation (Equation (5)):

(5) $$K=1000\cdot k=1000\cdot \frac{1.5\log\eta_{r}-1+\sqrt{1+\left(\frac{2}{c}+2+1.5\log\eta_{r}\right)1.5\log\eta_{r}}}{150+300c}$$

where *η_r_* = *η*/*η*_0_; *η* is the viscosity of the telomer solution; *η*_0_ is the viscosity of the pure auxiliary diluent (i.e., tetrahydrofurane); and *c* is the telomer concentration (g/cm^3^).

## 3. Results

### 3.1. Kinetics of the UV-Telomerization

**Figure 3:**
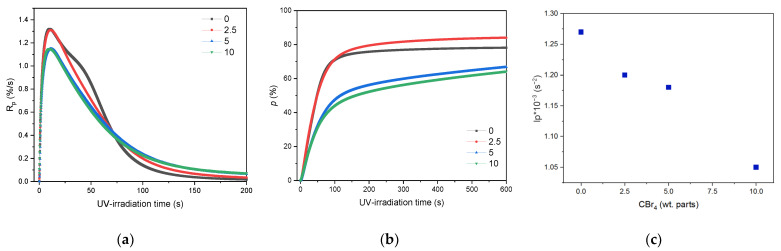
Reaction rate (**a**), double bond conversion (**b**), and photoinitiation index (**c**) during the UV-telomerization process of BA, AA, and ABP in the presence of various amount of CBr_4_ (I_0_ = 15 mW/cm^2^; 320–390 nm).

At the beginning, the influence of telogen content (2.5; 5 or 10 wt. parts) on the process of the UV-induced telomerization of the selected monomers system was investigated by the photo-DSC. The results are presented in Figure 3.

UV-telomerization differs fundamentally from photopolymerization in the concentration of radicals involved in the initiation stage. In photopolymerization, we have only one photoinitiator, and in telomerization, a two-component initiating system (PI-CBr_4_), with a large predominance of telogen. Although the concentration of primary radicals (resulting from the decomposition of the PI) is constant for all considered systems (0.6 mmol, Table 2), the concentration of radicals arising from the photolysis of CBr_4_ increases and is much higher than the concentration of primary radicals. Therefore, in the UV-telomerization process, primary termination (reaction of radicals with macroradicals) occurs much more often, and the reaction rate is independent of the telogen content, which is confirmed by the kinetic curves for the TS-5 and TS-10 systems (the same R_p_^max^). Additionally, in the case of two-component initiating systems, a generally observed phenomenon is the quenching of excited states by a quencher (telogen or monomer), which receives the excitation energy and dissipates it as heat. The studies showed that the photopolymerization (sample without telogen; black curve in Figure 3a) and telomerization processes with the lowest CBr_4_ content have almost the same R_p_^max^ (1.3%/s). However, when the telogen content increases (to 5 and 10 wt. parts), R_p_^max^ decreases only slightly (to 1.14%/s) and is the same for TS-5 and TS-10. It should also be noted that the photopolymerization process runs at a higher R_p_ than the telomerization process for a longer time (up to approximately 75 s), and then decreases rapidly, most likely due to a significant increase in the viscosity of the system and inhibition of the mobility of propagating macroradicals. In the case of UV-telomerization, shorter polymer (oligomer) chains are formed, which causes the viscosity of the system to be lower, and the chain propagation stage may take longer (the longer it is, the more telogen is in the system). Additionally, as the telogen concentration increases, the photoinitiation index (I_p_) of PI-telogen initiating systems decreases rapidly (Figure 3c). I_p_ means the overall ability to start the initiation reaction. Although it has already been proven that CBr_4_ itself undergoes photolysis and initiates the polymerization process itself, in the initiating system consisting of a radical photoinitiator and CBr_4_, the presence of telogen reduces the initiating abilities of such systems, which confirms the above statement that the reaction rate at a high concentration of radicals initiating the reaction becomes independent of the initiator concentration. Finally, it causes the lowest double bond conversion in this system, for TS-5 and TS-10 ca. 60% (Figure 3b). It is worth noting, however, that in the case of TS-2.5, the monomer conversion is as high as 82% and is slightly lower for the sample without telogen (77%).

### 3.2. The Physicochemical Properties of the Functional Acrylic Resins

**Figure 4:**
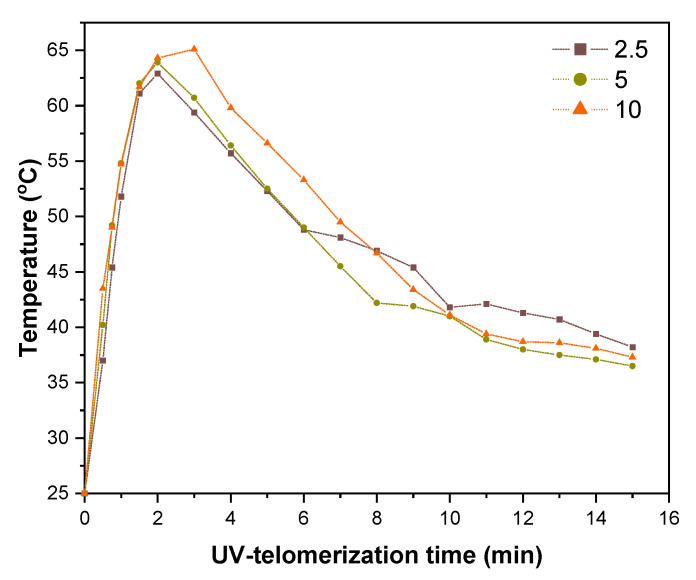
Temperature during the UV-telomerization process of BA, AA, and ABP with different amounts of CBr_4_.

The course of the UV-telomerization process in the glass reactor (at desired mixing speed of the reactants) was investigated by the registration of the maximum temperature (T_m_); the thermographs for the systems with different contents of telogen CBr_4_ (2.5; 5 or 10 wt. parts) are presented in Figure 4.

At the beginning, it should be mentioned that under the given photopolymerization conditions (UV dose), synthesis without the participation of telogen was not successful (i.e., sample TS-0). The system gelled rapidly after approximately 1 min of UV-exposure. This effect is also confirmed by the higher rate of photopolymerization (1.32%/s) than UV-telomerization demonstrated based on photo-DSC (Table 3).

**Figure 5:**
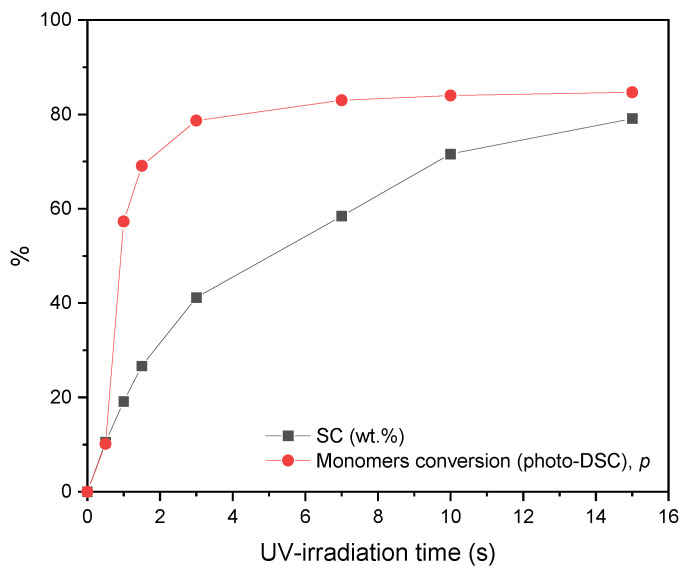
Comparison of *SC* values and double bond conversion (*p*) for the TS-2.5 sample depending on UV-exposure time.

The presented thermographs confirm that the higher CBr_4_ concentration generally slows down the UV-telomerization process because the recorded temperature peak occurred later (after 4 min of exposure for TS-10, while the temperature value was relatively high, i.e., T_m_ = 65 °C). The TS-2.5 system reached a slightly lower T_m_ value (62 °C). The T_m_ values do not correspond to the R_p_ values determined using the photo-DSC technique but confirm the phenomenon of quenching of excited states by a quencher (telogen), which receives the excitation energy and dissipates it as heat. Nevertheless, it should be noted that although the reactions in the glass reactor were carried out with the same qualitative and quantitative composition of reagents, in the same inert gas atmosphere, as well as the same initial temperature and UV-irradiation conditions, the assessment of the reactivity of the systems based on temperature recording differs from the reactivity of the systems determined based on photo-DSC research. This is also a result of the intensity of light absorbed in the large volume of the reaction mixture and mechanical mixing of reagents. During the photo-DSC test, we are dealing with a process in a thin layer (additionally carried out isothermally). The above is confirmed by a comparison of the results of the solids content value (*SC*) for an exemplary sample (TS-2.5) and the conversion of double bonds determined based on photo-DSC depending on the UV-irradiation time (Figure 5).

As can be seen, in the UV-telomerization process in a thin layer (photo-DSC), higher monomer conversions are achieved faster than in the bulk process using mechanical mixing in a glass reactor. This also translates into the final monomer conversion value (slightly lower for the reactor process). In turn, considering the influence of CBr_4_ concentration, the more telogen in the system, the higher the *SC* values for the obtained acrylic resins (from 79 to 85%, Table 3), contrary to the UV-telomerization in a thin layer (85 to 70%). This is because mechanical mixing prolongs the propagation stage because it allows the diffusion of macroradicals despite the increase in the viscosity of the system during irradiation. However, in the process carried out in a thin layer, the diffusion of macroradicals is impossible after reaching a certain critical viscosity of the system. It was revealed that a higher telogen content in the system has a beneficial effect on lowering its viscosity and a higher final *SC* results.

**Figure 6:**
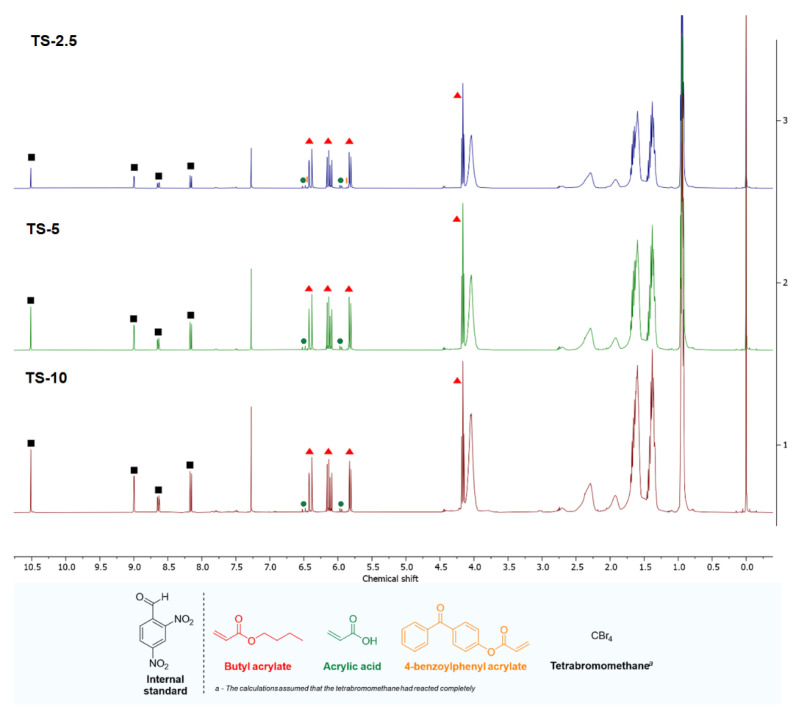
Stacked ^1^H NMR spectra of samples TS-2.5, TS-5, and TS-10.

**Figure 7:**
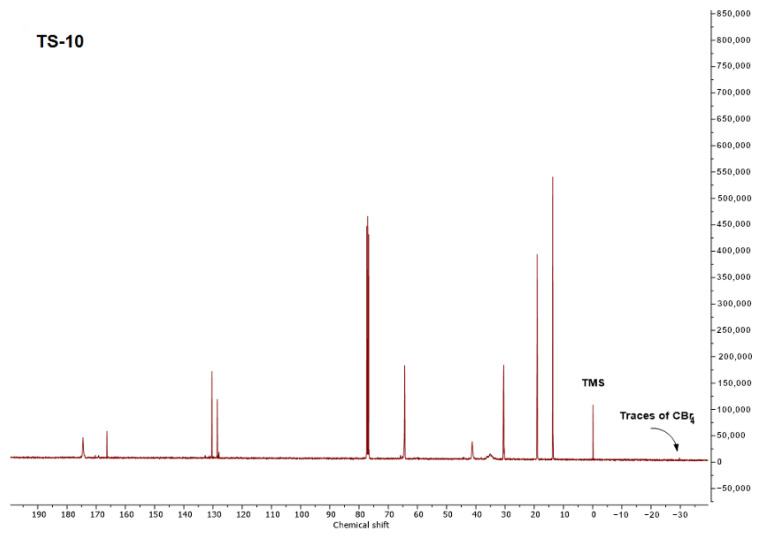
^13^C NMR spectrum of sample T-10.

The key issue in analyzing the course of telomerization is to demonstrate whether telogen has been incorporated into the structure of the resulting polymers/oligomers. For this purpose, nuclear magnetic resonance research was carried out. ^1^HNMR and ^13^C NMR spectra are shown for Figure 6 and Figure 7, respectively. The conversion of monomers and telogen was summarized in Table 4.

Due to the fact that CBr_4_ is invisible in ^1^H NMR spectra, ^13^C NMR spectra were performed to identify their possible presence in the sample. No signals from the above-mentioned compound were observed in the ^13^C NMR spectra of samples initially containing CBr_4_, or these signals were traces. Therefore, it was assumed in the calculations that these monomers had reacted completely. The conversion of individual monomers was very high (usually greater than 80%) and increased with the CBr_4_ content. Additionally, a higher concentration of telogen resulted in the complete incorporation of ABP into the structure of polymer chains, so photosensitive acrylate resins were obtained. However, the total conversion was high (from 79.5 to 88.3%), which also confirms the beneficial effect of telogen. It is also worth noting that the *SC* values correspond to the total conversion values determined using the NMR technique.

The molecular weights and the dispersity of the obtained telomeres are listed in Table 5. Based on these results, the prepared telomeres can be classified as polymers with low molecular weights and low dispersity (approx. 1.5), which indicates that the UV-telomerization process using CBr_4_ is well controlled and could be classified as a method for obtaining well-defined polymers (although not as precise as ATRP).

**Figure 8:**
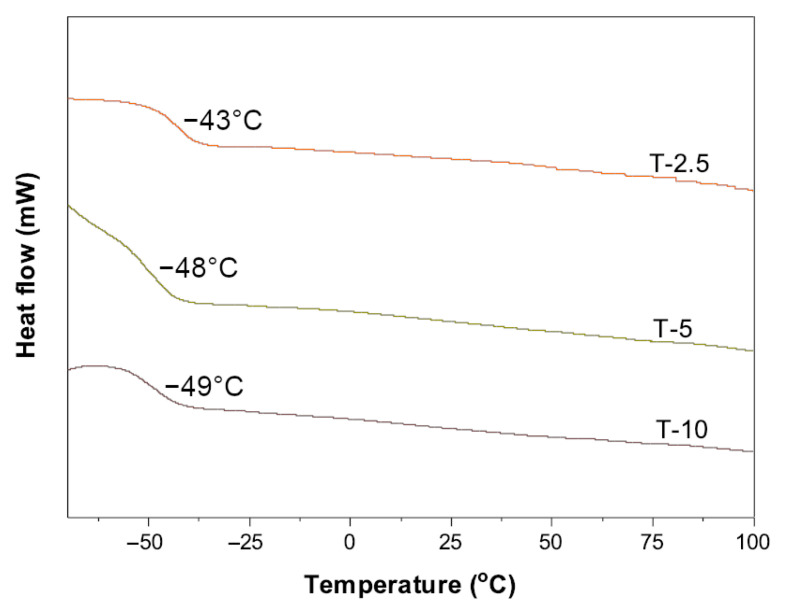
DSC thermograms of functional acrylic telomers.

As the telogen content in the system increases, the molecular weights decrease, but as previously shown in the case of large amounts of radicals (TS-5 and TS-10), the reaction rate is practically independent of telogen; therefore, no significant impact of telogen on the molecular weights of telomeres is observed (M_n_, M_w_, and K-values are close). It is known from the literature that CBr_4_ belongs to highly active telogens. In telomerization systems, chain transfer is an important factor in determining chain length (and so the molecular weights of telomers). Additionally, high rates of chain growth and chain transfer, relative to termination, provide products containing only negligible amounts of termination products. However, the dynamic viscosity of telomeric syrups decreases significantly. In terms of future applications of this type of resins (the possibility of using the TS-2.5 system to obtain self-adhesive glues has already been demonstrated [6]), the viscosity value (but also *SC*) is of key importance. These studies demonstrated the beneficial effect of increased telogen content on lowering the viscosity of resins (up to 6.3 Pa·s) and reducing the content of unreacted monomers (ca.12%). In terms of thermal properties, namely the glass transition temperature (Figure 8) of the telomeres themselves, it is similar for T-5 and T-10 (as well as the M_n_ and M_w_ values). It has been shown that an increase in telogen content reduces the glass transition temperature, which is related to the greater mobility of shorter polymer chains.

## 4. Conclusions

Acrylic resins functionalized in-chain and containing terminal Br atoms were prepared via the UV-telomerization process. The resins based on acrylic telomers syrups contained pendant carboxyl and benzophenone groups from functional monomers used to carry out the UV-telomerization process, i.e., acrylic acid and 4-acrylooxybenzophenone. The aim of this paper was to investigate the influence of telogen (tetrabromomethane, CBr_4_) on the kinetics of the UV-telomerization process and the physicochemical and thermal properties of the obtained resins. The main conclusions are as followed:

- A higher concentration of CBr_4_ in the UV-telomerization process no longer affects the maximum reaction rate but reduces the initiating capacity of the two-component initiating system.
- A higher telogen content has a positive effect on the increase in the total conversion of monomers and the reduction in the viscosity of the obtained resins.
- A high telogen content does not affect the molecular weights of telomeres but improves their unimodality (M_w_/M_n_ ca. 1.47); the glass transition temperature of telomeres does not change either.
- The UV-telomerization method of basic and functional monomers to obtain functionalized polymers is very promising because it is relatively simple and quick to perform (approx. 30 min). Additionally, it allows the acquisition of resins with a very low content of unreacted monomers (high-solid systems) and characterized by low dynamic viscosity.

## Figures and Tables

**Table 1:** Chemical structures of tested materials.

Monomers		
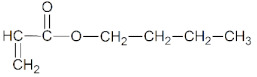	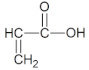	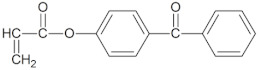
BA	AA	ABP
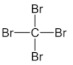	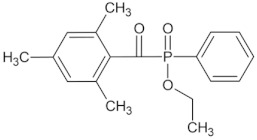	
	TPOL	

**Table 2:** Reaction mixtures used for the preparation of the functional acrylic telomers syrups.

Telomers Syrup	Monomers (wt. %)			Initiating System			
	BA	AA	ABP	CBr_4_ *		TPOL *	
				wt. Parts	mmol	wt. Part	mmol
TS-0	91.5	7.5	1.0	0	0	0.2	0.6
TS-2.5				2.5	7.5		
TS-5				5.0	15		
TS-10				10	30		

* per 100 g of monomers mixture.

**Table 3:** Comparison of *SC* values, double bond conversion (photo-DSC), and viscosity of the obtained FARs.

Sample	In Glass Reactor			In Thin Layer	
	*SC* (wt. %)	*T_m_* (°C)	t_Tm_ (min.)	*p* (%)	*R_p_^max^* (%/s)
TS-0	—	—	—	78	1.32
TS-2.5	79	62	2	85	1.31
TS-5	81	63	2	71	1.14
TS-10	85	65	4	70	1.14

**Table 4:** Monomers and telogen conversion in the telomer syrups (according to ^1^H NMR data).

Sample	Conversion (%)				
	BA	AA	ABP	CBr_4_	Total
TS-2.5	78.4	86.3	58.2	100	79.5
TS-5	80.7	87.5	100	100	82.7
TS-10	87.6	89.7	100	100	88.3

**Table 5:** Molecular weights and K-values of the telomers and dynamic viscosity of the telomers syrups.

Sample	M_n_ (g/mol)	M_w_ (g/mol)	Đ	*η* (Pa·s)	K-Value
TS-2.5	19,000	28,700	1.51	13.8	26.3
TS-5	17,000	25,000	1.47	7.3	18.1
TS-10	16,900	25,000	1.47	6.8	17.6

## Data Availability

The data presented in this study are available on request from the corresponding author.
